# Supplementary material for: Phase Behavior of Polydisperse Y-Shaped Polymer Brushes under Good Solvent Conditions
Source: Polymers (Basel). 2024 Mar 6;16(5):721. doi: 10.3390/polym16050721 (PMC10934012; doi:10.3390/polym16050721)
Supplement: Supplementary file 1 [file polymers-16-00721-s001.zip › polymers-2855499-supplementary.pdf]

SUPPLEMENTARY INFORMATION

# Phase Behavior of Polydisperse Y-Shaped Polymer Brushes under Good Solvent Conditions

Petr Fridrich and Zbyšek Posel *

*Department of Informatics, Faculty of Science, Jan Evangelista Purkyně University in Ústí nad Labem, 400 96 Ústí nad Labem, Czech Republic*

*Correspondence: zbysek.posel@ujep.cz

## Contents

**List of abbreviations** ... 2

**Distribution of chain lengths** ... 3

**Distribution of chains workflow** ... 4

**Dissipative particle dynamics** ... 5

**Additional results** ... 7

**References** ... 12

# List of abbreviations

Table 1: List of abbreviations used in the main text together with selected important parameters

| Abbreviation | Meaning |
|---|---|
| $PDI_A$ | Polydispersity index of homopolymer A |
| $PDI_B$ | Polydispersity index of homopolymer B |
| $N_A$ | Homopolymer A chain length |
| $N_B$ | Homopolymer B chain length |
| $f_A$ | Polymer chain length ratio: Ratio between homopolymer A chain length $N_A$ and total chain length $N_A + N_B$. |
| $P(N)$ | Schulz-Zimm distribution |
| $N_n$ | Number averaged chain length in Schulz-Zimm distribution |
| $N_w$ | Weight averaged chain length in Schulz-Zimm distribution |
| DPD | Dissipative particle dynamics |
| $\chi_{AB}$ | Flory-Huggins interaction parameter |
| $a_{AB}$ | Maximum repulsion between A/B homopolymer segments |
| $\sigma$ | Grafting density |
| $L$ | $x$ and $y$ dimension of the grafted surface |
| $L_z$ | z-dimension of the simulation box |
| $H$ | Brush height |
| $SF_{xy}$ | Lateral structure factor measured in $xy$ plane only |
| $M_S$ | Minkowski functional referring to the surface area covered by A homopolymer |
| $\rho_A$ | Density of A homopolymers |
| $\rho_B$ | Density of B homopolymers |
| PL | Perforated layer |

# Distribution of chain lengths

Chain length distributions from Schulz-Zimm [1,2] distribution calculated by Equation (1) in the main text. The Figure SI1 shows example for $f_A = 0.5$ and $N_n = 30$ for three different polydispersity values.

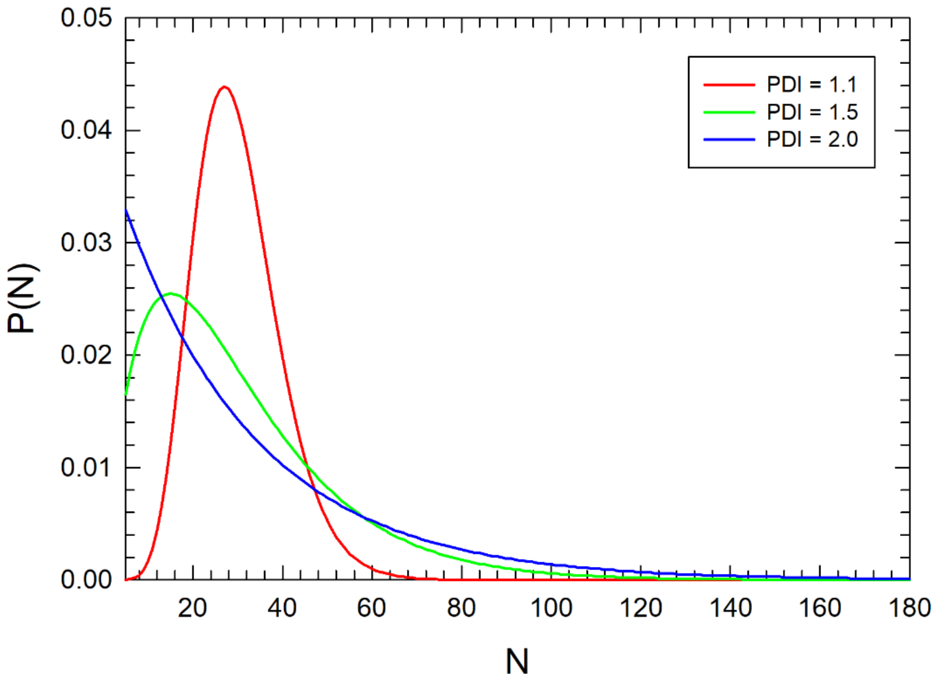


Figure S1*:* Distribution of chain lengths obtained from Schulz-Zimm distribution via Equation (1) in the main text. Red, green and blue line shows values for A homopolymer from Y-shaped brush with $N_n = 30$ at $f_A = 0.5$. The maximum chain length does not exceed 180 beads in one chain.

# Distribution of chains workflow

Simplified workflow for obtaining the distribution of chains that follows Schulz-Zimm distribution is presented below. In addition to polydispersity values for each homopolymer, $PDI_{A/B}$, and $f_A$ the input requires also the minimum and maximum chain length, total number of A/B chains and precision $\varepsilon$ that determines the maximum difference between selected $f_A$ and $f_A^*$ obtained from newly generated distribution. The usual value of $\varepsilon$ is equal to 0.001.

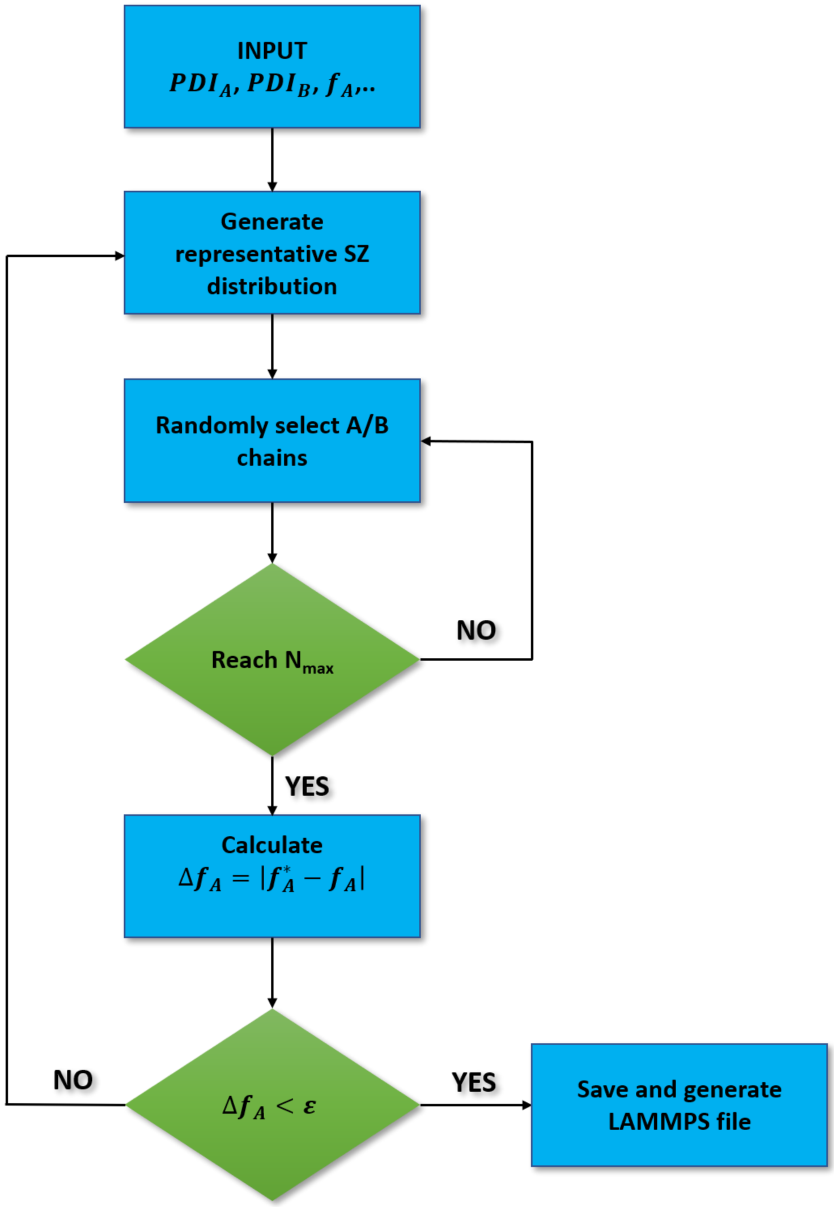


Figure S2: Simplified workflow used for generating the distribution of A/B chains in the brush. $PDI_{A/B}$ are polydispersity values for each homopolymer, $f_A$ is expected chain architecture and $f_A^*$ is actual chain architecture calculated from generated distribution. The precision $\varepsilon$, e.g. the maximum difference between expected and obtained chain architectures is set to 0.001.

## Dissipative particle dynamics

DPD has become a standard choice for modelling many phenomena on mesoscale level like self-assembly of polymers in melt [3-5], thin films [6,7], solutions [8] or in polymer-nanoparticle composites [9], just to mention a few. In DPD, a material is partitioned into beads where each bead can contain several atomistic particles or larger parts of material. Each bead is described by position $\boldsymbol{r}_i$, velocity $\boldsymbol{v}_i$, mass $m_i$ and interact with other beads by the force $\boldsymbol{F}_i$ written as a sum of three standard forces and additional forces that reflect bonds, bending etc. in complex molecules.

$$\begin{aligned}
\boldsymbol{F}_i &= \sum_{i\neq j} \boldsymbol{f}_{ij}^C + \sum_{i\neq j} \boldsymbol{f}_{ij}^D + \sum_{i\neq j} \boldsymbol{f}_{ij}^R + f_i^{bond} \\
\boldsymbol{f}_{ij}^C(\mathbf{r}_{ij}, a_{ij}) &= a_{ij}\left(1 - \frac{\mathbf{r}_{ij}}{r_c}\right)\boldsymbol{r}_{ij}^0 \\
\boldsymbol{f}_{ij}^D(\mathbf{r}_{ij}, \mathbf{v}_{ij}, \gamma_{ij}) &= \gamma_{ij}\,\omega^D(\mathbf{r}_{ij})(\mathbf{r}_{ij}\cdot\mathbf{v}_{ij})\boldsymbol{r}_{ij}^0 \\
\boldsymbol{f}_{ij}^R(\mathrm{r}_{ij}, \sigma_{ij}, \xi_{ij}) &= \sigma_{ij}\omega^C(\mathbf{r}_{ij})\frac{\xi_{ij}}{\mathbf{r}_v}\boldsymbol{r}_{ij}^0
\end{aligned} \tag{S1}$$

where $\boldsymbol{f}_{ij}^C(\mathbf{r}_{ij}, a_{ij})$ is conservative force, $\boldsymbol{f}_{ij}^D(\mathbf{r}_{ij}, \mathbf{v}_{ij}, \gamma_{ij})$ dissipative force and $\boldsymbol{f}_{ij}^R(\mathrm{r}_{ij}, \sigma_{ij}, \xi_{ij})$ random force, $\mathbf{r}_{ij} = \mathbf{r}_i - \mathbf{r}_j$, $\boldsymbol{r}_{ij}^0$ is the unit vector, $r_c$ is the cutoff distance, $a_{ij}$ is the maximum repulsion between two beads, $\mathbf{v}_{ij} = \mathbf{v}_i - \mathbf{v}_j$, $\gamma_{ij}$ and $\sigma_{ij}$ are the amplitudes of the dissipative and random force and $\xi_{ij}$ is the Gaussian random number with zero mean and unit variance, which is chose independently for each pair of beads. In addition, our DPD simulation contains also bonds described by harmonic spring force $f_i^{bond}$. Maximum repulsion between beads $a_{ij}$ is related to Flory-Huggins interaction parameter $\chi_{ij}$ [10] as

$$\frac{a_{ij} r_c}{k_B T} = \frac{a_{ii} r_c}{k_B T} + 3.27\chi_{ij} \tag{S2}$$

where $r_c$ is the cutoff distance, $k_B$ is the Boltzmann constant and $T$ is the thermodynamic temperature.

Parameters $\omega^D(\mathbf{r}_{ij})$ and $\omega^R(\mathbf{r}_{ij})$ in random and dissipative force are connected via dissipation fluctuation theorem [11] such as

$$\omega^D(\mathbf{r}_{ij}) = \left[\omega^R(\mathbf{r}_{ij})\right]^2$$
$$\sigma_{ij}^2 = 2\gamma_{ij}K_BT \qquad \text{(S3)}$$

and are typically chosen as

$$\omega^D(\mathbf{r}_{ij}) = \left[\omega^R(\mathbf{r}_{ij})\right]^2 \quad = \quad \left(1-\frac{r_{ij}}{r_c}\right)^2 \quad (r_{ij} < r_c)$$
$$\qquad\qquad\qquad\qquad\qquad = \qquad 0 \qquad (r_{ij} < r_c) \qquad \text{(S4)}$$

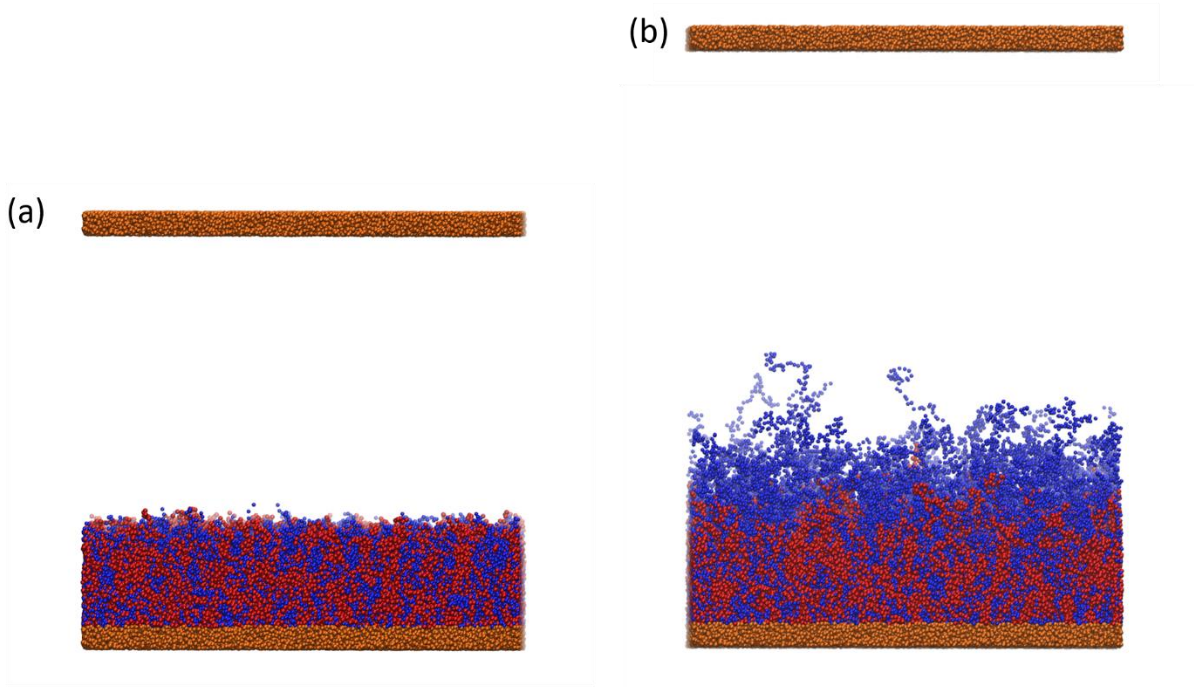


Figure S3: Front view of simulation box. (a) monodisperse system with $f_A = 0.5$ where both homopolymers have 30 beads in each grafted chain. (b) Polydisperse system with $f_A = 0.5$ and $PDI_A = 1.10$ and $PDI_B = 2.00$. Solvent is omitted from visualisation. Red color denotes A homopolymer, blue B homopolymer and orange stands for flat surface beads.

# Additional results

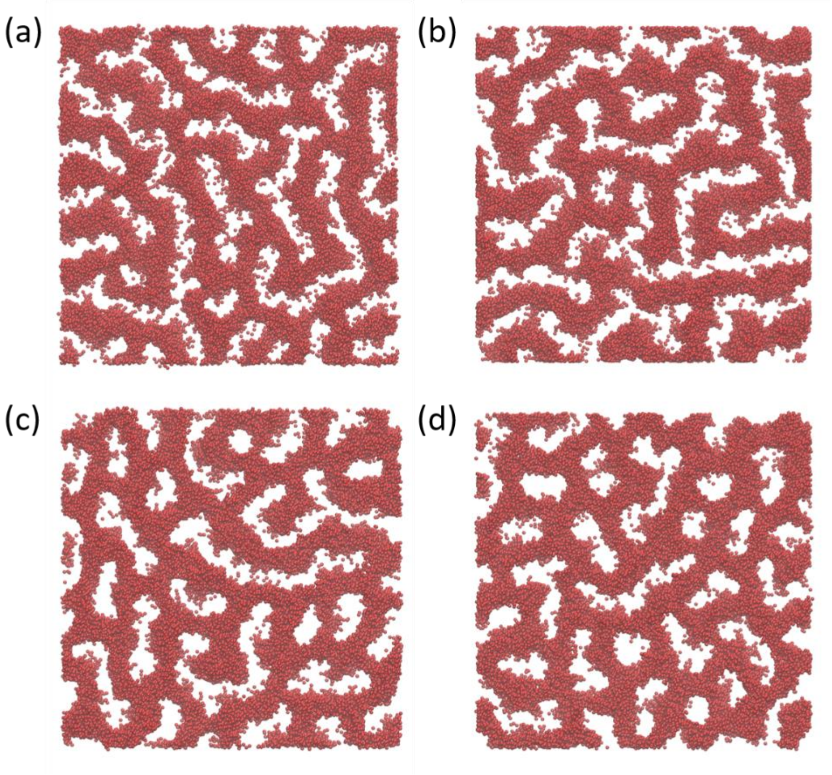


Figure S4: Top view of ripple structure for monodisperse and partially polydisperse systems at $f_A = 0.4$. Only monodisperse part of the Y-shaped brush is shown. Other is for clarity omitted from visualisation. (a) monodisperse system S1, (b) S2, (c) S3 and (d) S4.

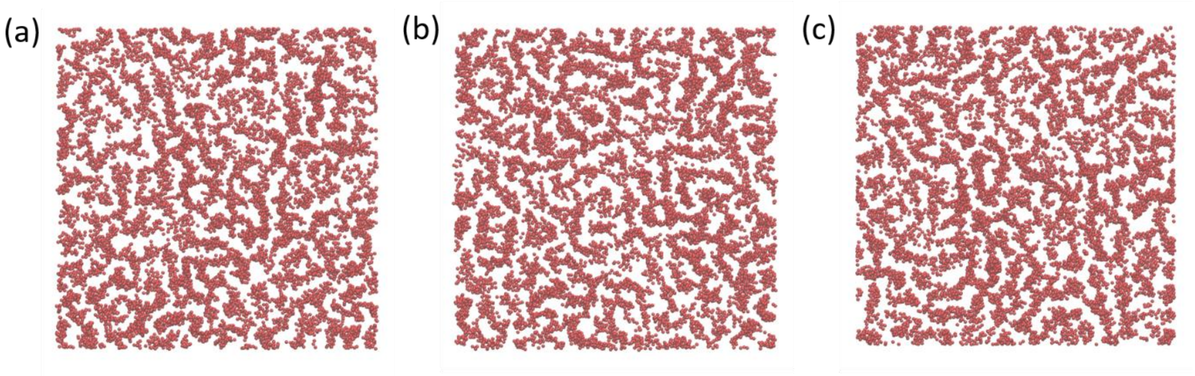


Figure S5: Top view of aggregates for monodisperse and partially polydisperse systems at $f_A = 0.1$. Only monodisperse part of the Y-shaped brush is shown. Other is for clarity omitted from visualization. (a) monodisperse system S1, (b) S2 and (c) S3. Remaining system S4 does not assemble to aggregates.

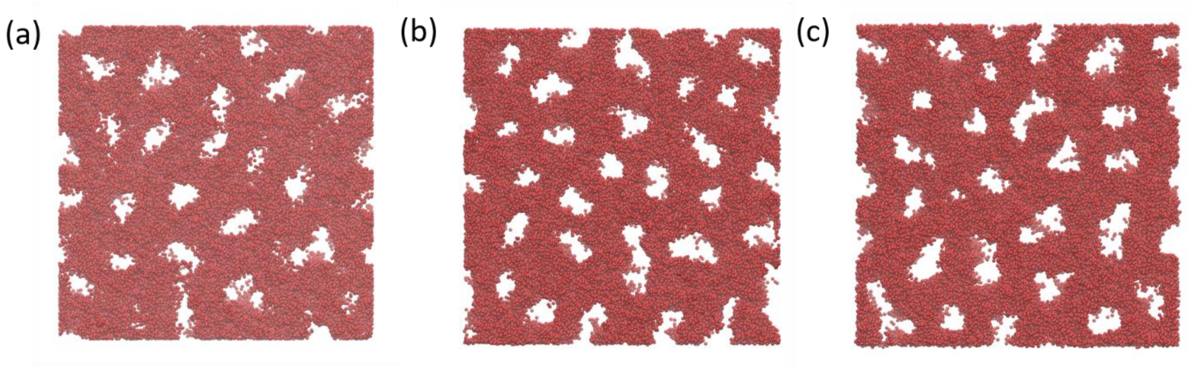


Figure S6: Top view of perforated layer for partially polydisperse systems at $f_A = 0.6$. Only monodisperse part of the Y-shaped brush is shown. Other is for clarity omitted from visualization. (a) S2 (b) S3 and (c) S4 system. Monodisperse system S1 does not form perforated layer.

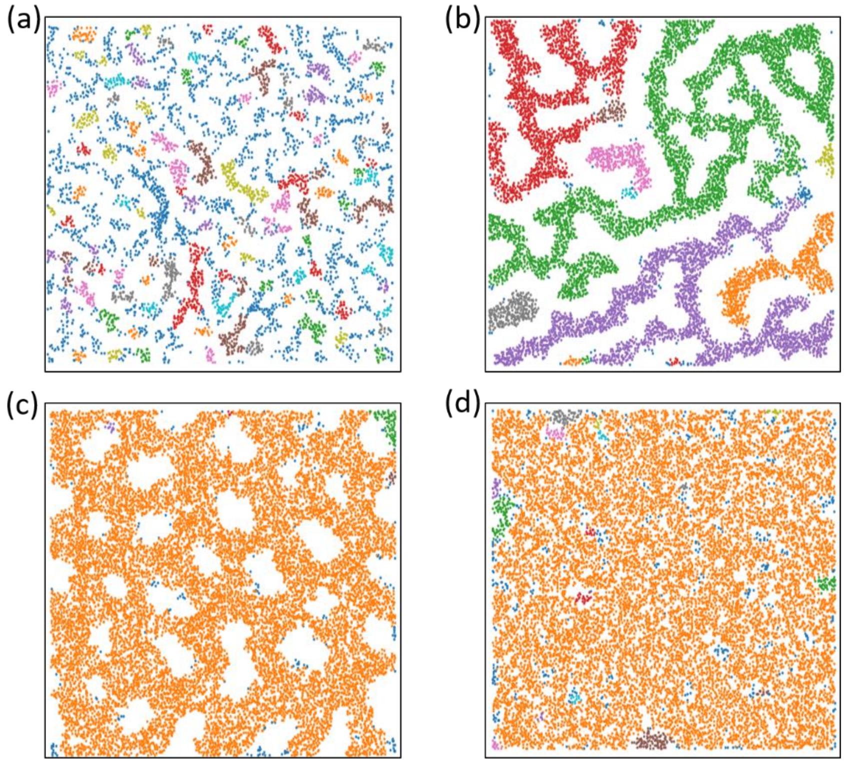


Figure S7: 2D configurational maps from DBSCAN used for proper identification of assembled structure. Each color represents one cluster in terms of DBSCAN parameters. Configurations are taken from system S3 in Figure 2 in the main text and (a) refers to clusters at $f_A = 0.1$, (b) to ripple structure at $f_A = 0.3$, (c) to perforated layer at $f_A = 0.6$ and (d) to compact layer at $f_A = 0.9$, respectively.

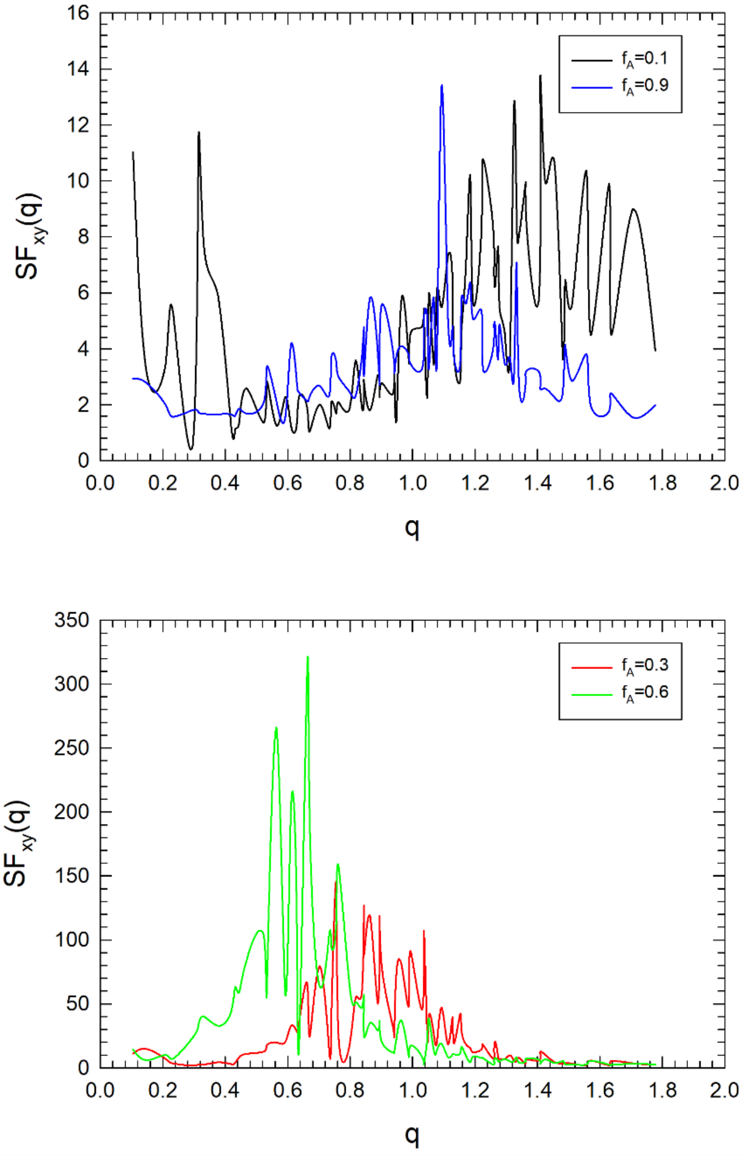


Figure S8: Lateral structure factor for configurations taken from system S3 in Figure 2 in the main text. $f_A = 0.1$ refers to clusters, $f_A = 0.3$ to ripple structure, $f_A = 0.6$ to perforated layer and $f_A = 0.9$ to compact layer, respectively. Due to different scales, the data were separated into two figures. Profiles were calculated for A homopolymer only.

(a)

(b)

(c)

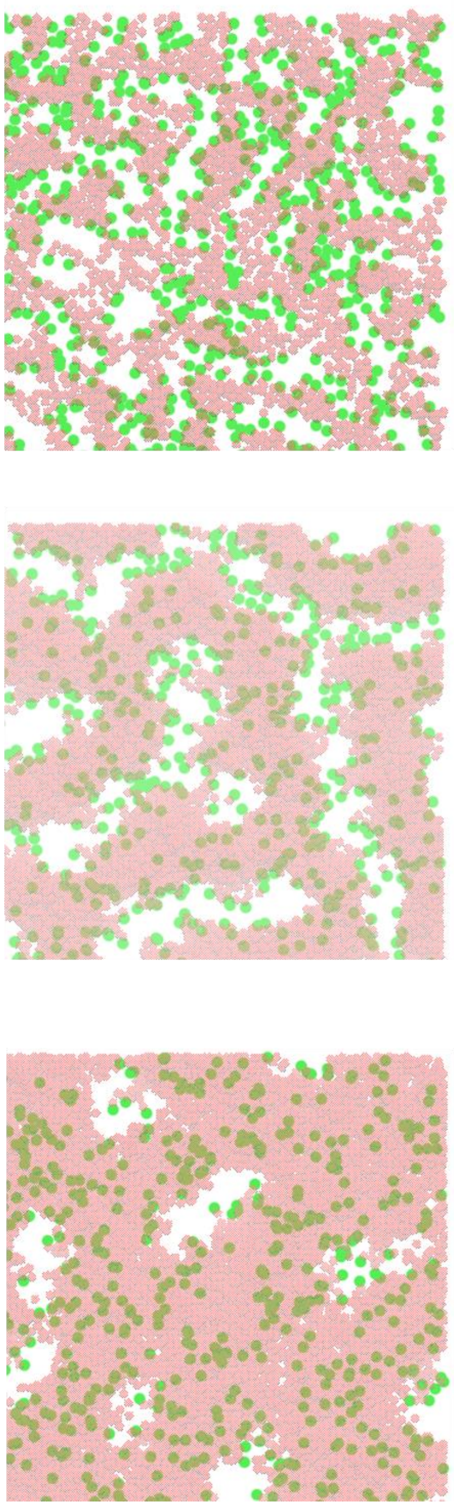

Figure S9: Visualization of relationship between position of brush grafting points (green) and assembled structure (red) for partially polydisperse system S2 ($PDI_A = 1.0, PDI_B = 1.1$). For clarity, only A homopolymer is displayed, other components are omitted from visualization. Moreover, to discern individual brush grafting points only $30r_c \; x \; 30r_c$ sample of the surface is shown that represent right upper quarter of the full surface. (a) shows aggregates from Figure SI5b, (b) shows ripple phase from Figure SI4b and (c) shows perforated layer from Figure SI6a, respectively.

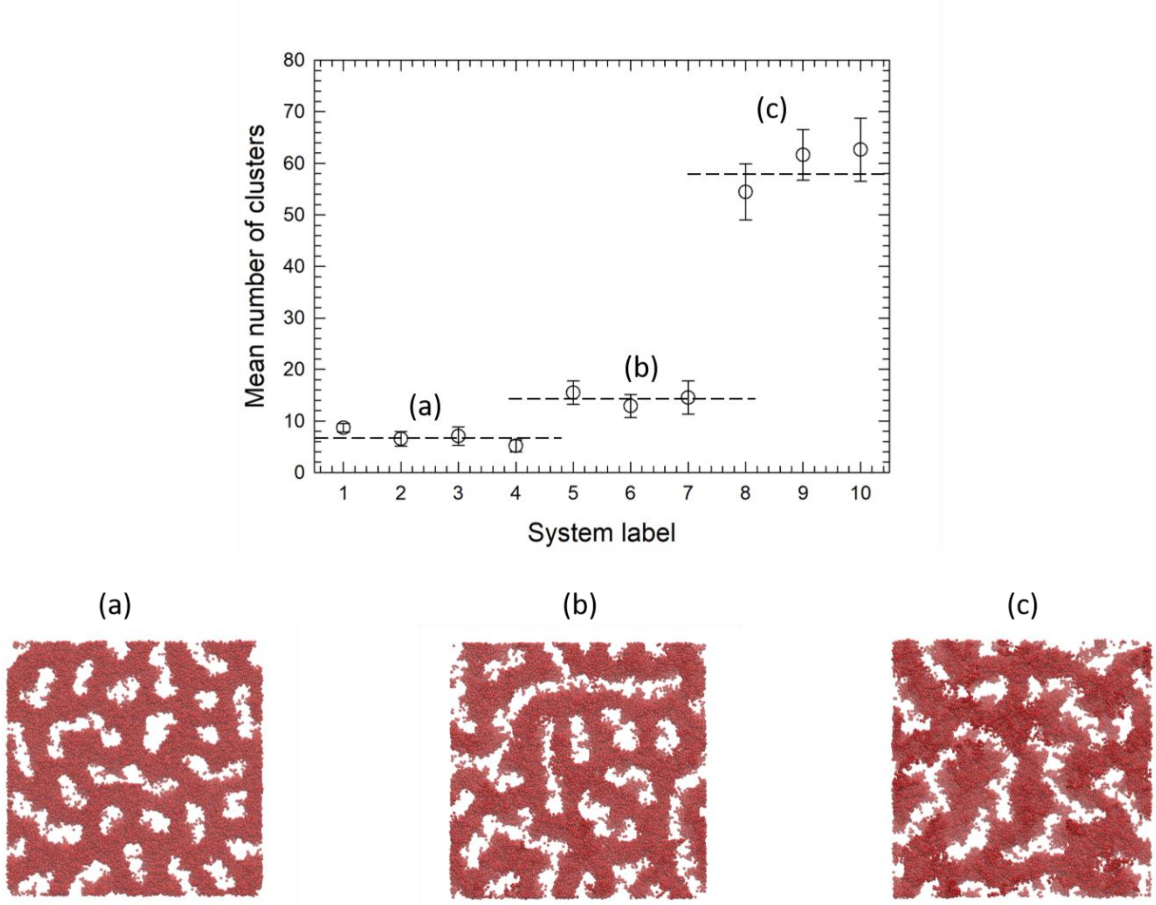


Figure S10: Top: Mean number of clusters for all systems at $f_A = 0.5$. Group (a) refers to monodisperse system and partially polydisperse systems with $PDI_A = 1.0$. Group (b) refers to fully polydisperse system where $PDI_A = 1.1$ and (c) refers to fully polydisperse systems where $PDI_A = 1.5$. Corresponding configurations are shown at the bottom of the figure where red spheres represent only homopolymer A while homopolymer B is for clarity omitted from visualisation.

# References


1. Zimm, B.H. Apparatus and Methods for Measurement and Interpretation of the Angular Variation of Light Scattering - Preliminary Results on Polystyrene Solutions. *J Chem Phys* **1948**, *16*, 1099-1116.
2. Patil, R.R.; Turgman-Cohen, S.; Srogl, J.; Kiserow, D.; Genzer, J. On-Demand Degrafting and the Study of Molecular Weight and Grafting Density of Poly(methyl methacrylate) Brushes on Flat Silica Substrates. *Langmuir* **2015**, *31*, 2372-2381.
3. Gavrilov, A.A.; Kudryavtsev, Y.V.; Chertovich, A.V. Phase diagrams of block copolymer melts by dissipative particle dynamics simulations. *J Chem Phys* **2013**, *139*.
4. Posel, Z.; Rousseau, B.; Lisal, M. Scaling behaviour of different polymer models in dissipative particle dynamics of unentangled melts. *Mol Simulat* **2014**, *40*, 1274-1289.
5. Posel, Z.; Posocco, P.; Lisal, M.; Fermeglia, M.; Pricl, S. Highly grafted polystyrene/polyvinylpyridine polymer gold nanoparticles in a good solvent: effects of chain length and composition. *Soft Matter* **2016**, *12*, 3600-3611.
6. Guskova, O.A.; Seidel, C. Mesoscopic Simulations of Morphological Transitions of Stimuli-Responsive Diblock Copolymer Brushes. *Macromolecules* **2011**, *44*, 671-682.
7. Posel, Z.; Posocco, P. Tuning the Properties of Nanogel Surfaces by Grafting Charged Alkylamine Brushes. *Nanomaterials-Basel* **2019**, *9*.
8. Posel, Z.; Svoboda, M.; Colina, C.M.; Lísal, M. Flow and aggregation of rod-like proteins in slit and cylindrical pores coated with polymer brushes: an insight from dissipative particle dynamics. *Soft Matter* **2017**, *13*, 1634-1645.
9. Karatrantos, A.; Clarke, N.; Kroger, M. Modeling of Polymer Structure and Conformations in Polymer Nanocomposites from Atomistic to Mesoscale: A Review. *Polym Rev* **2016**, *56*, 385-428.
10. Groot, R.D.; Madden, T.J. Dynamic simulation of diblock copolymer microphase separation. *J Chem Phys* **1998**, *108*, 8713-8724.
11. Español, P.; Warren, P.B. Perspective: Dissipative particle dynamics. *J Chem Phys* **2017**, *146*.
